# Case of Placenta Prævia

**Published:** 1854-04

**Authors:** J. K. Mason


### *Case of Placenta Præevia.* By J. K. Mason, M. D.

In reading the different works on midwifery, it will, I think, occur somewhat painfully to the mind of every young practitioner, that the treatment of that most formidable of all the accidents to which child-bearing women are exposed, viz: placenta prævia, is far less clearly defined than might be wished ; nor can it ever be otherwise, for it is the inherent consequence of the very nature of the difficulty, in which the danger is so imminent, and the tendency to death so strong and rapid, as in some instances almost to preclude the possibility of any treatment whatever.

As in all critically dangerous cases, different modes of treatment have been advocated and adopted, and each has had its successes to record and its followers to panegyrize.

Some authors tell us that our reliance must be on the use of the tampon until the os uteri be sufficiently dilated or dilatable to permit the operation of turning, without lacerating the womb by forcing an entrance and thereby destroying the woman; but this direction is too vague to be of much practical use; nor could any man with a heart in his body submit to obey it with a fellow creature bleeding to death before him. No, he must act to accomplish a great good; he must nerve himself to incur some risk. He may, it is true, be a little too soon, but he is between Scylla and Charybdis. It were worse to be too late.

On the other hand, some distinguished authorities object to the use of the tampon, considering it insufficient and even dangerous. An eminent European professor has recently advocated the practice of separating the whole of the placenta and removing it as soon as the os uteri is sufficiently dilated or dilatable. But with deference I must confess I am not convinced that we have gained much by the suggestion, for the poor sufferer might still bleed to death long before the aforesaid requisite degree of dilatation or dilatability had taken place.

These gentlemen bring forward cases, each to prove the superior efficacy of his own mode of practice, and there can be no doubt but that each mode has occasionally succeeded, for such accidents left entirely to nature have sometimes by her unaided efforts alone, been brought to a happy termination.

My own conviction is, that in the practice of medicine, midwifery or surgery, all exclusive modes of treatment are unphilosophical and absurd; that in unavoidable hæmorrhage especially, the treatment which in one case would enable the unfortunate woman to overcome the frightful danger which threatens her, might in another be utterly useless, or even hurry on the dreaded catastrophe.

In relation to the treatment of placenta prævia I do not presume to offer any theory or even a single practical novelty, convinced as I am, that all the hobby horses will occasionally share the same fate and be found terribly lame at the bed side; but having recently treated such a case successfully, for the sufferer, though bloodless, blanched and nervous to an extreme degree, is slowly recovering, being now in her 21st day, I will relate the circumstances as they occurred; state how, when and why I acted, and leave my readers to judge the practice by the best test we have, the result:

Feb. 12th, 1854. Was called to Mrs. H——, then carrying her thirteenth child. I found her in bed, very much alarmed by some sudden gushes of blood attended with pain, though not of a severe nature. This condition of things, I was informed, had continued for nearly two hours. When I reached her the bleeding had entirely ceased. On making some enquiry I found that Mrs. H. considered her pregnancy advanced to about the end of the eighth month, and on making an examination per vaginam I was satisfied by the condition of the neck of the womb that her calculation was a correct one. At this time she complained a little of pain, that her head felt dull and loaded, with a sense of aching and stiffness, as she described it, in the eyelids; the pulse was rapid and full, and there was a feeling of heaviness and oppression in the chest; there was also considerable epigastric uneasiness.

These symptoms made me fearful of an attack of puerperal convulsions, and I immediately took some sixteen ounces of blood from her arm, with the effect of giving great relief, and after prescribing an anodyne, left her.

On my visit the next morning found her very much improved, pulse good, respiration free, head lighter and unoppressed, countenance natural and mind free from anxiety; but at midnight she had another sudden attack of hæmorrhage unaccompanied by pain or uneasiness. It stopped, however, as suddenly as it came on, and she remained tranquil and comfortable.

Three nights after, on the 19th, she had another attack of the same nature—the loss of blood greater than before—without the slightest pain or disposition on the part of the uterus to take on expulsive action.

I now felt convinced that I had to contend with a case of presenting placenta, but to what extent, whether partial or complete, it was impossible to form an estimate. On this occasion, as formerly, the flooding after a few gushes suddenly ceased, and I

left her with direction to keep perfectly quiet, and send for me on the slightest renewal of the cause of alarm.

I heard no more of her until the 23d. About one o'clock, A. M., I was roused from my bed to visit her, hurried to the house, and on entering her room found her looking tolerably well though much alarmed. She informed me that she had had a tremendous gush of blood. I immediately examined the bed, and was much relieved by finding that she had not lost a drop, but that the membranes had given away and that it was the discharge of the liquor amnii which had caused the fright. Still not the slightest pain or contraction of the uterus.

Made an examination per vaginam and found the cord presenting. This circumstance satisfied me that if the placenta presented it could be but partial, and I hoped that regular contraction would shortly ensue and terminate this hitherto discouraging case, without further difficulty. There was not the slightest tinge of blood in the amniotic discharge, and the cord pulsated strongly, but the os uteri was so slightly dilated and the probability of a natural delivery so good that the attempt to turn would have been perfectly unjustifiable.

After waiting an hour without perceiving the slightest manifestation of change, I returned to my house, which was in the immediate neighborhood, desiring them to send for me on the slightest appearance of uterine action. In a very short time I was hastily summoned, and on reaching my patient found that she had been seized with a sudden and tremendous flooding. She was pale, gasping, almost pulseless, and partially convulsed. Brandy was freely administered; and now satisfied that another such hæmorrhage would be fatal, I applied the tampon and sent for my friend Dr. Hollingsworth, resolved, should he approve, to turn and deliver by the feet as soon as the poor woman's strength should be sufficiently rallied to justify the operation.

The Doctor was with me in a very short time, and, on seeing the state of the case, agreed with me, that the child must be turned, and the uterus emptied at all hazards, as soon as some degree of reaction would warrant the attempt. We gave her brandy in repeated doses, and resolved to wait and watch for the favorable time. After the lapse of two hours, finding that there were no pains, that the pulse did not improve in force, and that there was some slight loss of blood through the tampon, and fearing internal hæmorrhage, we determined to act without further delay; accordingly forty grains of an infusion of ergot were administered, after which I removed the plug, and passing my hand cautiously into the vagina, I found the os tincæ open to about the size of a dollar, but lax; the uterus being perfectly passive, caused, I presume, by the great loss of blood. My hand, therefore, passed, without much resistance, into the organ; the child lay to the left side, nearly in the first position of Baudaloque, and the placenta could be distinctly felt on the right side, extending from the os, covering about a third of its disc, and stretching up towards the fundus.

The left foot of the child was first brought into the vagina; this Dr. H. secured with a fillet, and I proceeded in search of the right. The ergot, or the hand, or perhaps both—I have considerable faith in the manual-stimulation—had now produced some uterine action, which rapidly increased, so much so, indeed, that the introduction of the hand was much more difficult; having, however, secured the right foot, it was brought into the vagina, and a dead child brought away without much further difficulty—Dr. H. supplying the patient freely with stimuli, and keeping up a steady pressure on the womb, so as to cause it, by its tonic contraction, as it were, to follow up the exit of its contents.

There was no hæmorrhage from the time that I first introduced my hand. The placenta was easily removed, and, a most gratifying circumstance, the patient's strength seemed to be rather improved than exhausted by the operation.

The treatment of this case since delivery it would be useless and tiresome to repeat. Suffice it to say that it has been chiefly addressed to the end of calming nervous irritation, which has been very considerable.

The recovery has especially been retarded by great irritability of stomach, which, as it for some days precluded the possibility of throwing much nourishment into the system, has necessarily kept her weak and depressed.

She has neither tympanitis nor pain, nor tenderness in the region of the abdomen or pelvis, but is occasionally harassed by a succession of tremors, unaccompanied by any sensation of cold—an irregular, nervous action, I imagine produced by the great deficiency of the stimulating portion of the blood, viz., the red globules.

She is now taking quinine and iron, with milk punch, beef tea, &c., for diet; and occasionally an enema containing opium.

